# Nocardia Otomastoiditis in a Chemotherapy-Induced Neutropenic Patient With Diffuse Large B-cell Lymphoma: Successful Medical Management

**DOI:** 10.7759/cureus.101265

**Published:** 2026-01-10

**Authors:** Ifra Nasir, Faisal Sultan, Khalid Javed, Aun Raza, Salma Abbas

**Affiliations:** 1 Internal Medicine/Infectious Diseases, Shaukat Khanum Memorial Cancer Hospital and Research Centre, Lahore, PAK; 2 Radiology, Shaukat Khanum Memorial Cancer Hospital and Research Centre, Lahore, PAK; 3 Medicine, Shaukat Khanum Memorial Cancer Hospital and Research Centre, Lahore, PAK

**Keywords:** chemotherapy-induced neutropenia, diffuse large b-cell lymphoma, immunocompromised host, nocardia otomastoiditis, trimethoprim-sulfamethoxazole

## Abstract

*Nocardia *species are aerobic, filamentous, weakly acid-fast, Gram-positive bacteria. They cause opportunistic infections, particularly in patients receiving chemotherapy or corticosteroids. Otologic involvement is extremely rare, with only a few cases of *Nocardia* otomastoiditis reported worldwide.

We report a 31-year-old woman with gastric and pancreatic diffuse large B-cell lymphoma who presented with fever and a one-week history of worsening purulent discharge from the left ear during chemotherapy-induced neutropenia. She had a 15-year history of intermittent otorrhea, previously treated with oral amoxicillin-clavulanate with transient improvement. On examination, she was febrile, with granulation tissue and purulent discharge in the left external auditory canal. Laboratory investigations confirmed neutropenia. Gram stain and modified Ziehl-Neelsen staining of the ear swab revealed beaded, branching, partially acid-fast filamentous organisms. Culture identified a *Nocardia *species susceptible to trimethoprim-sulfamethoxazole (TMP-SMX). Computed tomography (CT) imaging demonstrated otomastoiditis without bony erosion or intracranial extension.

The patient was treated with a brief empiric course of intravenous imipenem, followed by eight weeks of oral TMP-SMX, resulting in the complete resolution of clinical symptoms and significant radiologic improvement.

This case highlights the importance of considering *Nocardia* infection in patients with chronic or refractory otorrhea, particularly in the setting of chemotherapy-induced neutropenia. An early microbiologic diagnosis and focused therapy can lead to complete healing without surgery or long-term use of multiple drugs.

## Introduction

*Nocardia *species are aerobic, filamentous, Gram-positive bacteria found in soil, organic debris, and water worldwide. Human infection typically occurs via inhalation or direct inoculation and predominantly affects immunocompromised hosts, including those receiving chemotherapy, corticosteroids, or organ transplantation [1]. Otologic infection due to *Nocardia *is exceptionally uncommon, with only a few reports of *Nocardia *otitis media or mastoiditis in the literature [2-11].

Clinical manifestations vary with the site of infection and immune status, with pulmonary, cutaneous, and central nervous system involvement being the most frequent [12,13]. Diagnosis is often delayed because of nonspecific clinical features and the slow growth of the organism in culture, leading to advanced disease and poor outcomes [12]. Early recognition and targeted antimicrobial therapy are therefore crucial, especially in patients with hematologic malignancies who experience transient immunosuppression during chemotherapy.

To our knowledge, this is among the very few reported cases of *Nocardia *otomastoiditis in a patient receiving chemotherapy for diffuse large B-cell lymphoma (DLBCL) and one of the few cases successfully managed with early microbiologic diagnosis and medical therapy alone. This case highlights the importance of considering atypical pathogens in refractory otologic infections and contributes to the limited evidence on managing localized *Nocardia *infections in immunocompromised patients.

## Case presentation

A 31-year-old woman with a known diagnosis of gastric and pancreatic DLBCL presented on April 9, 2025, with a one-day history of fever and a one-week history of worsening purulent discharge from her left ear. She reported experiencing intermittent left-ear discharge for 15 years, suggestive of a chronic otologic process. Previous treatment with oral co-amoxiclav provided only transient relief. She denied using topical ear drops or other local therapies. No prior ear cultures or imaging studies had been performed.

She had no history of diabetes mellitus, chronic liver disease, chronic kidney disease, or prior ear surgery. She was a nonsmoker, did not consume alcohol, and reported no occupational or environmental exposure to soil or organic material.

The patient received her first cycle of R-CHOP (rituximab, cyclophosphamide, doxorubicin, vincristine, prednisolone) chemotherapy on March 31, 2025. Her otologic symptoms worsened shortly after starting chemotherapy, likely due to chemotherapy- and corticosteroid-induced immunosuppression.

On presentation, the patient was febrile (38.5°C), tachycardic (heart rate: 140 beats/min), and hypotensive (90/65 mmHg). Otoscopy of the left ear showed purulent discharge and granulation tissue within the external auditory canal, with tenderness over the mastoid region; the tympanic membrane was intact. No neurological deficits were observed. The right ear and systemic examination, including chest auscultation, were unremarkable.

Laboratory investigations showed profound neutropenia (absolute neutrophil count=0 cells/µL) and markedly elevated C-reactive protein, consistent with neutropenic sepsis. Renal and hepatic function remained normal throughout hospitalization, allowing the continuation of trimethoprim-sulfamethoxazole (TMP-SMX) without dose adjustment. The absolute neutrophil count recovered by April 13, 2025, along with defervescence and clinical improvement (Table 1).

**Table 1:** Key laboratory parameters during hospitalization µL: microliter; mg/dL: milligrams per deciliter; mg/L: milligrams per liter; U/L: units per liter

Parameter (units)	Value	Reference range
Absolute neutrophil count (cells/µL)	0	1500-8000
C-reactive protein (mg/L)	101.83	<5
Serum creatinine (mg/dL)	0.4	0.6-1.2
Blood urea nitrogen (mg/dL)	7.56	7-20
Alanine aminotransferase (U/L)	16	7-56
Aspartate aminotransferase (U/L)	13	10-40
Total bilirubin (mg/dL)	0.15	0.1-1.2

**Figure 1:**
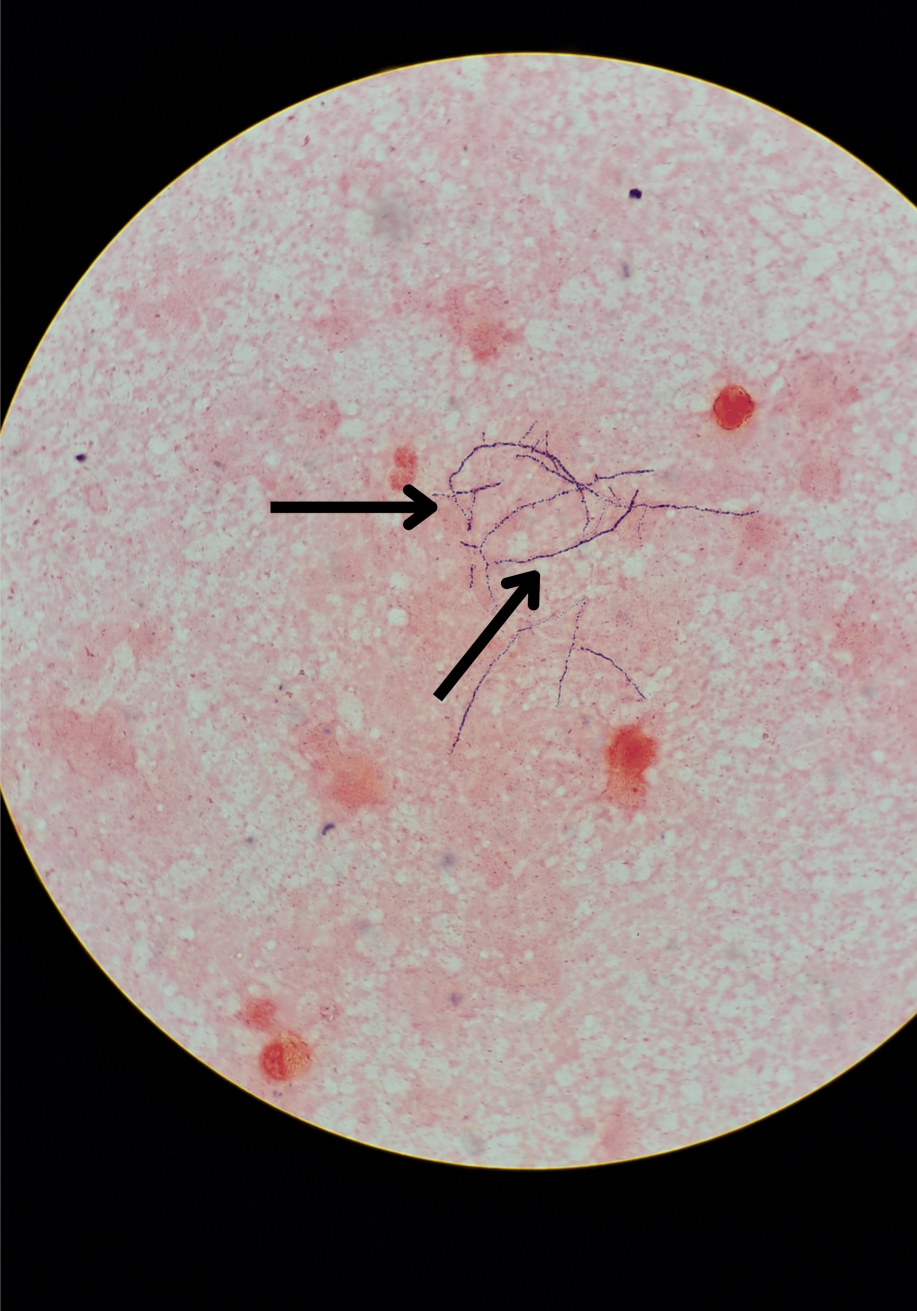
Gram-stained smear of the left-ear swab Filamentous, beaded, Gram-positive branching rods consistent with *Nocardia *species (black arrows), viewed under oil immersion at ×1000 magnification.

**Figure 2:**
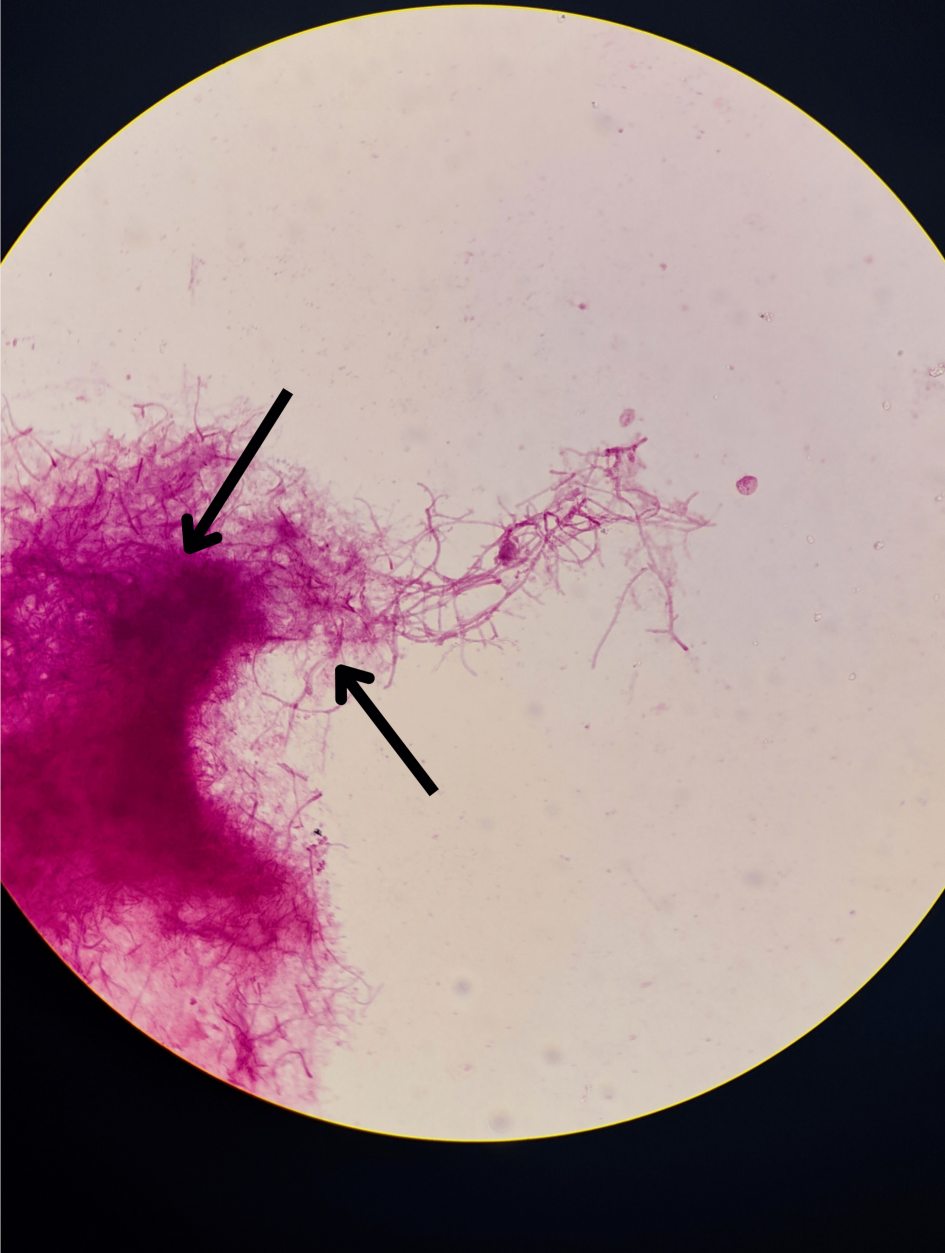
Modified Ziehl-Neelsen (Kinyoun) stain of the left-ear swab Thin, branching, filamentous, partially acid-fast organisms (black arrows) consistent with *Nocardia *species, viewed under oil immersion at ×1000 magnification.

**Figure 3:**
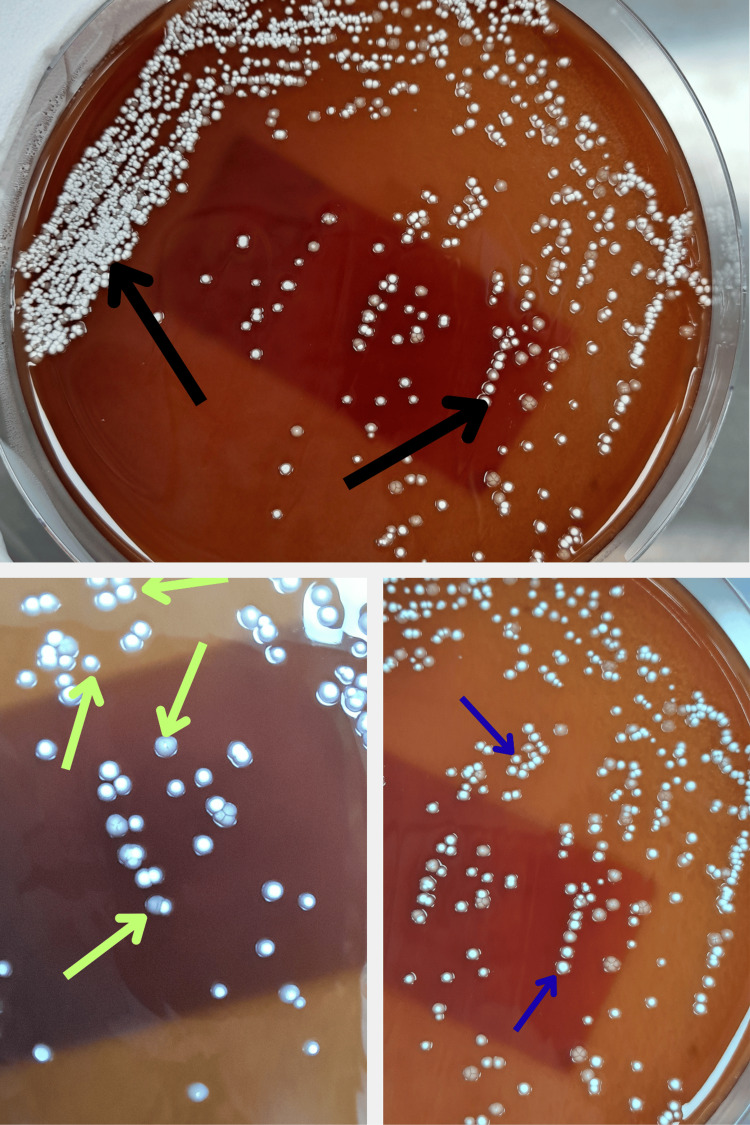
Nocardia colonies on blood agar *Nocardia* growth at 37°C after 72 hours of aerobic incubation, showing dry, chalky-white colonies (arrows) with a rough, breadcrumb-like surface.

Two sets of blood cultures obtained on April 9, 2025, were sterile. A left-ear swab taken the same day showed Gram-positive, beaded, branching rods on Gram stain (Figure 1) and partially acid-fast filamentous organisms on modified Ziehl-Neelsen stain (Figure 2), consistent with *Nocardia* species. Definitive identification of *Nocardia *from the left-ear swab was confirmed by culture on day 5 (Figure 3).

Antimicrobial susceptibility testing showed susceptibility to TMP-SMX, ceftriaxone, tobramycin, and linezolid. Molecular species identification was not performed.

**Figure 4:**
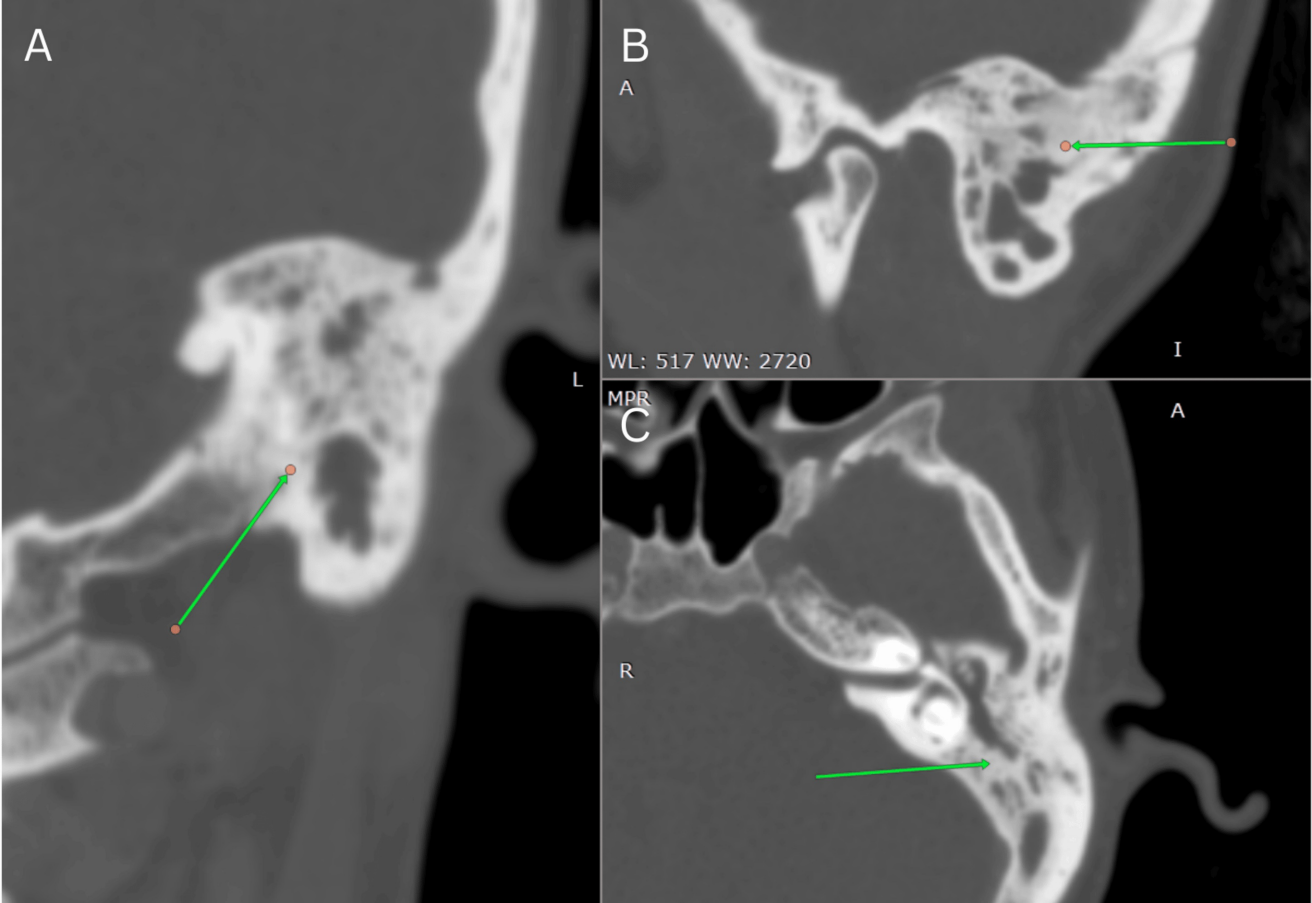
High-resolution computed tomography of the left temporal bone (April 2025) (A) Sagittal/oblique view (coronal reformat): complete soft tissue opacification of the left external auditory canal and middle ear cleft (green arrows), consistent with otomastoiditis. (B) Axial view: dense inflammatory soft tissue filling the mastoid air cells (green arrow), consistent with otomastoiditis. (C) Coronal view: soft tissue within the middle ear and mastoid antrum abuts the tegmen tympani (mastoid roof) with preserved bony margins.

Computed tomography (CT) of the brain and temporal bone (April 10, 2025) revealed the complete opacification of the left mastoid air cells and middle ear cavity with soft tissue thickening around the auditory ossicles but no bony erosion or intracranial extension, consistent with otomastoiditis (Figure 4).

A chest radiograph (April 14, 2025) showed clear lung fields, a normal cardiac silhouette, and no evidence of consolidation or effusion. 

**Figure 5:**
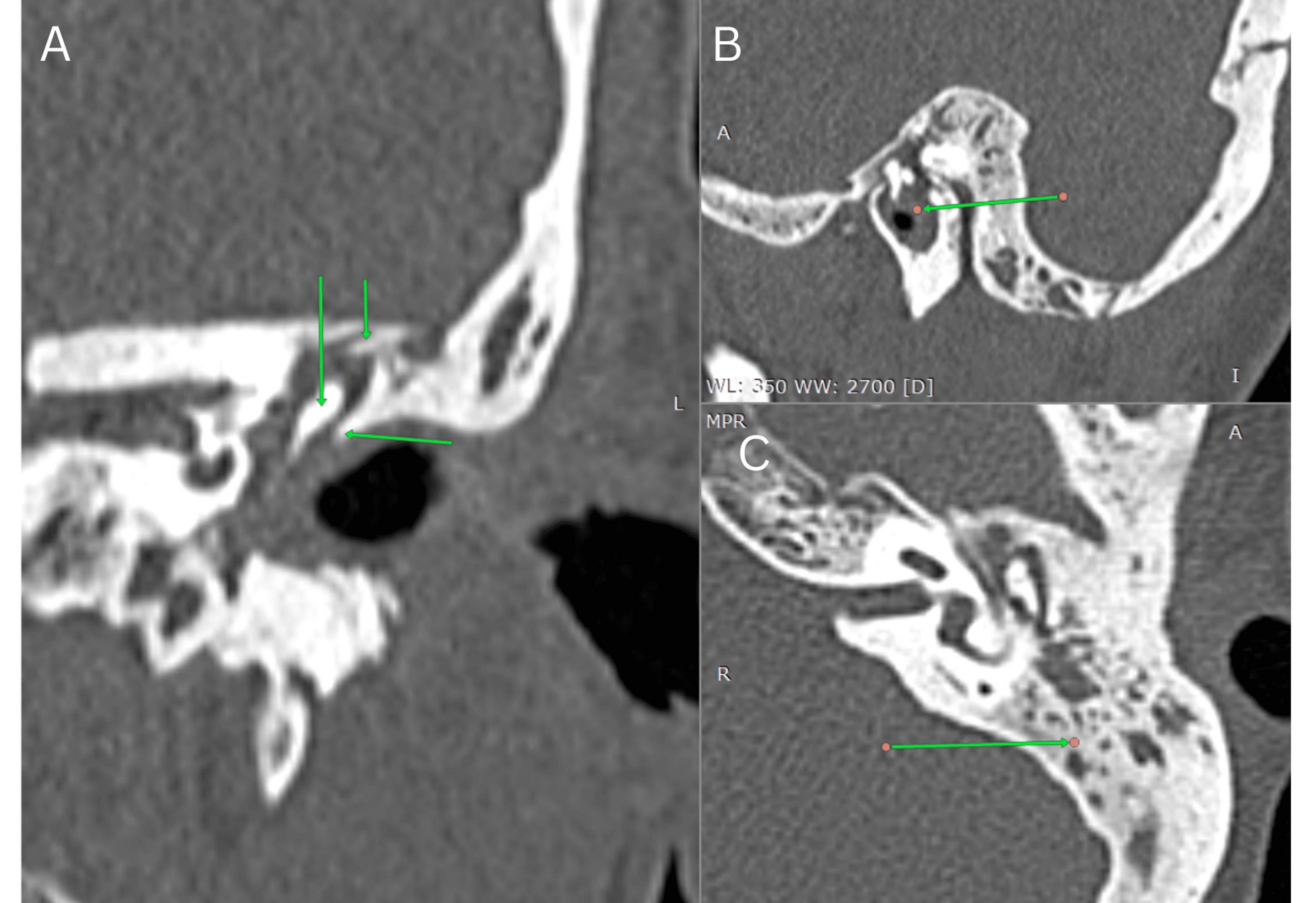
Follow-up high-resolution computed tomography of the temporal bone (May 2025): interval improvement after medical therapy (A) Sagittal/oblique view: marked reduction of soft tissue opacification in the left middle ear and mastoid, showing re-aeration (green arrows). (B) Axial view: near-complete clearing of mastoid air cells: minimal residual soft tissue. (C) Coronal view: reduced middle ear and mastoid effusion, along with enhanced visibility of the tympanic cavity and mastoid antrum.

A repeat CT of the temporal bone (May 3, 2025) showed interval improvement with minimal middle ear and mastoid effusion and chronic inflammatory changes (Figure 5).

**Figure 6:**
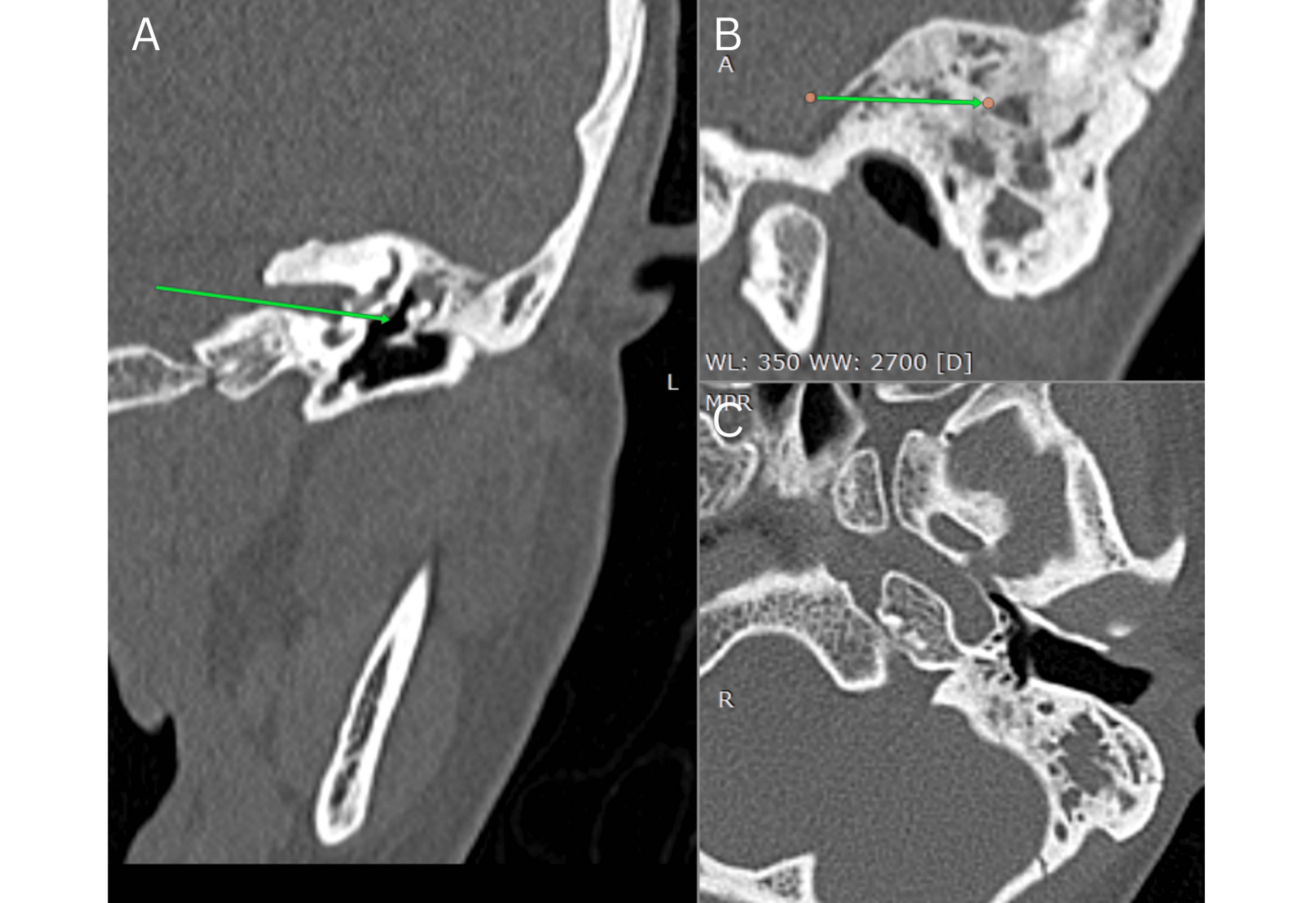
High-resolution computed tomography of the temporal bone (September 2025): near-complete resolution (A) Sagittal/oblique view: near-complete resolution of inflammatory changes in the middle ear and mastoid; green arrow highlights fully re-aerated cavities. (B) Axial view: mastoid air cells are fully re-aerated with well-delineated bony septa. (C) Coronal view: normal aeration of the middle ear and mastoid with intact bony margins; minimal residual mucosal thickening reflects prolonged radiologic cure.

Follow-up CT (September 2, 2025) showed the near-complete resolution of the effusion (Figure 6).

Empiric therapy was started with intravenous imipenem (500 mg every six hours) and oral TMP-SMX (160 mg/800 mg twice daily, approximately 15 mg/kg/day of the trimethoprim component), covering Gram-negative and atypical pathogens, including *Nocardia*. After neuroimaging excluded intracranial involvement, imipenem was stopped, and TMP-SMX monotherapy was continued for eight weeks based on susceptibility results and clinical response.

The patient improved rapidly, with defervescence within five days and resolution of otorrhea and mastoid tenderness. No surgical intervention was needed.

Follow-up CT of the temporal bone (September 2, 2025) showed marked resolution of mastoid and middle ear effusion. She completed eight weeks of cotrimoxazole and remained afebrile and asymptomatic.

At the six-month follow-up (October 20, 2025), she stayed well, with no recurrence of otorrhea or systemic symptoms.

## Discussion

*Nocardia *species are uncommon opportunistic pathogens that predominantly affect immunocompromised hosts, including those receiving chemotherapy, corticosteroids, or organ transplantation [1,8]. Clinical presentation varies according to the site of infection and the degree of immune suppression. Although pulmonary, cutaneous, and central nervous system involvement are most common, otologic disease remains exceedingly rare. The literature documents only a handful of *Nocardia *otitis or mastoiditis cases worldwide and even fewer among patients undergoing chemotherapy. Our case adds to this limited evidence base by describing the successful treatment of localized otomastoiditis in a patient with DLBCL who developed transient neutropenia during R-CHOP chemotherapy.

The pathogenesis of *Nocardia* infection in immunocompromised hosts has been well characterized. Palomba et al. [1] emphasized that most cases occur in patients with hematologic malignancies, transplants, or prolonged corticosteroid exposure. Similarly, *Steinbrink *et al. [13] reported that immunocompromised individuals are more likely to experience disseminated disease and higher mortality than immunocompetent hosts. In our patient, transient chemotherapy-induced neutropenia may have predisposed to the localized infection of the mastoid and middle ear. The absence of dissemination or recurrence after neutrophil recovery suggests that transient immunosuppression may lead to organ-confined nocardiosis when promptly recognized and treated.

In contrast, we were able to diagnose our case early by using Gram and modified Ziehl-Neelsen staining on the mastoid discharge, which showed filamentous, branching, weakly acid-fast organisms that were compatible with *Nocardia*. Early microbiologic confirmation helped target TMP-SMX treatment, resulting in complete clinical remission and radiologic improvement, reflecting the resolution of mastoid and middle ear inflammation on follow-up CT, without the need for surgical intervention. This result highlights the necessity of including *Nocardia *infection in the differential diagnosis of refractory otitis or mastoiditis, especially in immunocompromised or neutropenic individuals. 

The duration of treatment for nocardiosis is not definitive. Conventional guidelines advocate for six months of treatment for localized disease and up to one year for disseminated infection [12]. Recent research, however, suggests that shorter treatment periods may be enough for non-disseminated illness in patients experiencing immunological recovery. In our instance, eight weeks of TMP-SMX resulted in total remission without recurrence, consistent with findings by Marsh et al. [4] and Subha and Raman [5]. Consequently, therapy decisions ought to be informed by illness localization, organism susceptibility, and the host's immune condition.

Our results corroborate the findings of Martínez-Barricarte [14], who suggested that isolated nocardiosis may occur in the setting of temporary rather than persistent immunological failure. Palomba et al. [1] likewise underscored that prompt diagnosis and proper treatment are essential for positive outcomes. Najafizadeh et al. [11] documented a similar instance of *Nocardia* pansinusitis in persistent lymphocytic leukemia, underscoring the diagnostic difficulties associated with nonspecific otolaryngologic manifestations in immunocompromised individuals and the importance of prompt culture-based confirmation to avert dissemination.

Although surgical drainage is generally necessary for advanced mastoiditis, this case illustrates that early, localized infections can be adequately treated with targeted medicinal therapy alone. This is different from the examples Li et al. [2] and Gkrinia et al. [3] talked about, where difficulties in the brain that needed surgery happened because they weren't recognized right away.

To put our findings in context, Table 2 shows a summary of previously reported otologic *Nocardia *infections, showing how different host immunological statuses, pathogen species, management techniques, and outcomes can be. This comparison underscores the distinctiveness of our situation and advocates for prompt microbiological assessment and focused treatment in immunocompromised individuals.

**Table 2:** Comparative summary of reported otologic Nocardia infections This table includes patient characteristics, underlying conditions, clinical presentation, diagnostic method, pathogen species, management, and outcomes. DLBCL: diffuse large B-cell lymphoma; TMP-SMX: trimethoprim-sulfamethoxazole; CSOM: chronic suppurative otitis media; CT: computed tomography; MRI: magnetic resonance imaging; CNS: central nervous system

Author/year	Age (years)	Sex	Immune status	Clinical presentation and imaging findings	Other localization	Diagnostic method	*Nocardia *species	Treatment and duration	Outcome	Key clinical point
Li et al., 2023 [2]	45	M	Immunocompetent (healthy adult)	Otalgia, hearing loss, mastoid swelling; CT showed otomastoiditis	None	Culture and 16S rRNA PCR	Nocardia farcinica	Mastoidectomy + TMP-SMX for 12 weeks	Recovered	First reported surgically treated *N. farcinica* otomastoiditis
Gkrinia et al., 2025 [3]	45	F	Immunocompetent (healthy adult)	Otalgia, facial palsy, mastoid tenderness; MRI showed an epidural abscess	Epidural extension	Tissue culture	Nocardia farcinica	Mastoidectomy + TMP-SMX for 12 weeks	Recovered	Severe intracranial extension in an immunocompetent host
Marsh et al., 2017 [4]	62	M	Immunocompromised (multiple myeloma on chemotherapy)	Recurrent otitis media, mastoid pain; CT showed mastoid cavity infection	None	Culture of biopsy specimen	*Nocardia *spp.	Mastoidectomy + TMP-SMX + amikacin for 6 weeks	Recovered	*Nocardia* mastoiditis in hematologic malignancy
Subha and Raman, 2004 [5]	45	M	Immunocompromised (HIV-positive)	Chronic otorrhea, mastoid tenderness; CT showed mastoid opacification with a petrous apex abscess	None	Culture of aspirate	*Nocardia *spp.	Mastoidectomy + TMP-SMX for 10 weeks	Recovered	First reported mastoid *Nocardia* infection in HIV
Chawla et al., 2014 [6]	10	F	Immunocompetent (healthy pediatric)	Chronic suppurative otitis media with postauricular swelling; MRI showed cortical venous thrombosis	CNS venous thrombosis	Culture from ear discharge	*Nocardia *spp.	Mastoidectomy + TMP-SMX for 12 weeks	Recovered	Pediatric CSOM complicated by intracranial venous thrombosis
Lawler et al., 2014 [7]	8	M	Immunocompromised (HIV-positive)	Otorrhea with postauricular abscess; CT showed mastoid destruction	None	Pus culture	*Nocardia *spp.	Surgical drainage + TMP-SMX for 6 weeks	Recovered	First African pediatric case of *Nocardia* mastoiditis
Forrett-Kaminsky et al., 1991 [8]	38	M	Immunocompromised (AIDS)	Otorrhea and mastoid pain	None	Culture from the middle ear fluid	Nocardia asteroides	TMP-SMX + surgical drainage	Recovered	Early AIDS-associated *Nocardia* otitis media
Salvo Gonzalo et al., 1996 [9]	32	F	Immunocompetent (healthy adult)	Acute otitis media with tympanic membrane perforation	None	Culture	Nocardia asteroides	TMP-SMX	Recovered	Acute* Nocardia* otitis media in a healthy adult
Cox et al., 1986 [10]	12	M	Immunocompetent (post-surgical pediatric)	Brain abscess following mastoid surgery	CNS abscess	Culture	Nocardia asteroides	IV TMP-SMX for 8 weeks + surgery	Recovered	Postsurgical CNS dissemination
Present case (Nasir et al., 2025)	31	F	Immunocompromised (chemotherapy for gastric DLBCL)	Ear pain, otorrhea, hearing loss; CT showed otitis media with effusion	None	Culture from ear *swab*	*Nocardia *spp.	IV imipenem + oral TMP-SMX for 8 weeks	Complete recovery	One of the few oncologic cases successfully cured with medical therapy alone

This case highlights several important clinical aspects. First, *Nocardia* infection should be considered in refractory otologic disease, particularly in immunocompromised or neutropenic patients receiving chemotherapy or corticosteroids. Second, early microbiologic identification using modified acid-fast staining and culture is essential for prompt management. Third, TMP-SMX remains an effective first-line therapy; monotherapy is often sufficient for treating localized infections. Finally, multidisciplinary coordination between infectious disease, oncology, and otolaryngology teams is vital for improving outcomes.

Limitations include the single-patient design and absence of molecular species identification, limiting generalizability. Nevertheless, given the rarity of otologic *Nocardia* infection, it contributes to meaningful clinical evidence supporting the early recognition and individualized therapy in immunocompromised hosts.

## Conclusions

Early suspicion and culture-based confirmation of *Nocardia* can prevent progression and may obviate the need for surgery, even in immunocompromised hosts. *Nocardia* should be considered in the differential diagnosis of chronic or refractory otorrhea, particularly in patients receiving chemotherapy or corticosteroids. Prompt microbiologic evaluation, exclusion of dissemination, and prompt initiation of TMP-SMX can result in complete recovery without surgical intervention. Vigilance for atypical pathogens in refractory otologic infections, especially during chemotherapy-induced neutropenia, is essential to ensure timely, organ-confined management and improved outcomes.
